# A sudden creatinine increase: A case report

**DOI:** 10.11613/BM.2022.011002

**Published:** 2022-02-15

**Authors:** Bernhard Strasser, Sebastian Strasser, Josef Tomasits

**Affiliations:** 1 Kepler University Hospital Linz, Institute of Laboratory Medicine, Linz, Austria; 2 Medical University of Vienna, Vienna, Austria

**Keywords:** cystatin C, creatinine, estimated glomerular filtration rate, case report

## Abstract

Creatinine and estimated glomerular filtration rate (eGFR) are first-line laboratory parameters in the diagnosis of various renal diseases. In recent decades, cystatin C (cysC) has furthered the laboratory repertoire regarding renal status assessment and has been implemented in many clinical guidelines. Accordingly, with the establishment of cysC as a renal routine biomarker, further opportunities for assessing eGFR have been attained. Nevertheless, various limitations are still associated with cysC and creatinine analysis. Preanalytical errors could cause false results in both biomarkers. In our case, we were confronted with implausibly elevated creatinine levels due to preanalytical errors.

## Introduction

Biomarkers, such as cystatin C (cysC), creatinine, and estimated glomerular filtration rate (eGFR), represent the commonly approved laboratory basis program in the assessment of patients’ renal status. Comparing creatinine and cysC, different physiological functions and metabolisms could be distinguished, which further explain slight differences in their use as laboratory biomarkers. Naturally, both molecules are predominantly eliminated by the kidneys.

Creatinine is the end product of muscle catabolism. The biosynthesis of creatine originates from the amino acids arginine, glycine, and methionine in an initial enzymatic reaction. Creatine is a nitrogenous organic acid preferentially generated in the liver but also in the kidneys and pancreas. It is then further processed to creatinine; in the skeletal and heart muscles, creatine is converted to creatine phosphate, which acts as an important energy source for muscular contractions. In addition, in the muscle cells, creatine is irreversibly converted to creatinine, which is finally eliminated by the kidneys. Beyond that endogenous production, creatinine can also be alimentarily ingested because it is specifically found in dietary proteins (*1*).

In contrast to creatinine, cysC is a ubiquitously expressed molecule of nucleated cells with specific immune-modulatory functions. As a cysteine protease inhibitor, it is essentially responsible for protecting cells from protease degradation. CysC is freely filtered and mostly reabsorbed in the proximal tubule; consequently, cysC represents another suitable renal biomarker, although its international standardization is yet to be definitively accomplished (*2*, *3*).

## Laboratory analyses

Here, we report a 61-year-old patient who is essentially healthy according to his age. Although his medical records showed no indicators of renal impairment, he was interested in an outlined preventive medical examination and asked for an analysis of relevant biomarkers that reflect on his kidney function. We consequently analysed his creatinine and cysC and obtained non-pathological results. One hour later, we decided to control our results by taking a new sample for reanalysis and surprisingly received conspicuously elevated creatinine levels in combination with cysC results within the reference interval as it is demonstrated in Table 1.

**Table 1:** Pre- and postprandial laboratory assessment of creatinine and cysC

**Baseline**	**Results**	**RI**
Creatinine (µmol/L)	88	59-103
CysC (nmol/L)	62.92	45.69-71.16
Postprandial		∆%
Creatinine (µmol/L)	132	50
CysC (nmol/L)	63.66	/
RI – reference interval. ∆% – relative delta change between baseline and postprandial analysis.		

## Further investigations

We primarily suspected haemolysis, lipaemia, or bilirubin as potential interference factors because many studies have already described artificially increased creatinine results in that context (*4*-*6*). However, macroscopic inspections could not corroborate that suspicion, and the haemolysis, icterus, and lipaemia indices yielded very low counts in both samples.

## What happened

At lunchtime, our patient had previously eaten boiled beef for dinner. The second blood sample was taken too early after food intake (one hour postprandial) and altered the creatinine analysis without influencing the cysC concentration in repeat testing.

## Discussion

The analytical variability of creatinine is relatively high compared to cysC. Because cysC is continuously released from nucleated cells, it is almost invariably independent of muscle mass, gender, age, and ethnicity (*7*). In our case, food ingestion was the incriminated confounder that led to the false pathological creatinine results. Previous studies have already demonstrated this effect in depth. For reproducible reasons, this analysis error was described in different analytical laboratory systems, not only in the Jaffe method but also in approaches using alkaline picrate or enzymatic methods. Importantly, creatine-rich foods such as beef or fish are the main contributors to such falsely elevated results because creatine is converted to creatinine during the cooking process (*8*, *9*). Interestingly, the impact of creatine intake on creatinine mismeasurement seems to be higher in patients with chronic kidney diseases. Nair *et al.* further demonstrated a creatinine peak after two hours in healthy individuals and after four hours in patients with advanced chronic kidney disease after consuming a cooked meat meal (*10*). Jacobsen *et al.* also reported that when six volunteers ate a beef-containing meal, their subsequent creatinine levels did not recover to their initial creatinine levels within 20 hours (*11*). However, limitations of their study included small sample size, unknown methodology for creatinine analysis, and unknown renal status of the volunteers (specifically, whether or not any participants had impaired renal function). These factors are important to consider when questioning the amount of time, it takes to regain uninfluenced results. In addition, individuals’ constitutions may differ with regards to absorption and metabolization rates. Suggesting a general timeframe post food ingestion would thus be non-definitive, and it would be more rational to comply with fasting recommendations to avoid the detection of artificially higher creatinine levels (*11*).

Translating those findings into our case, we concluded that the second postprandial analysis was performed significantly too early in order to generate correct creatinine results. The working group on the preanalytical phase of the European Federation of Clinical Chemistry and Laboratory Medicine (EFCC) recommended a food fasting period of 12 hours and alcohol abstinence of 24 hours before blood samples are taken.

It has also been postulated that a worldwide standardization of blood sampling and patient preparation, respectively, is necessary. For example, specialists in laboratory medicine have been encouraged to elaborate on acceptance criteria related to fasting issues and to contemplate sample rejections in cases of non-compliance (*12*).

The International Federation of Clinical Chemistry and Laboratory Medicine (IFCC) and the EFCC published a consensus paper on performance specifications for the extra-analytical phase. In this paper, they focused on harmonizing quality indicators of the laboratory process and identified inappropriate or unintelligible test requests as important quality indicators of the preanalytical phase (*13*). Correspondingly, Mrazek *et al.* published a report on overutilization as a relevant preanalytical problem (*14*). Among other things, this problem can be traced back to non-adherence with regards to retesting intervals. This study group suggested that specialists in laboratory medicine should proactively participate in the preanalytical phase of test ordering to prevent inappropriate test requests. They also published strategies and solutions to reduce test-overutilization (*i.e.*, starting with educational feedback, interpretive comments and automated flags, gate-keeping strategies over to implemented algorithms and reflex criteria, and the establishment of diagnostic management teams) (*14*).

In our case, apart from non-compliance to an appropriate fasting period, a further preanalytical error existed in that the second creatinine analysis was not required. According to the recommendations listed above, we reanalysed creatinine levels on the next day under strict adherence to the fasting period and obtained results close to the baseline creatinine measurement of the day before (96.8 µmol/L) prior to food ingestion. This finding supported the suggestion of alimentary influence on consecutive mismeasurement of creatinine.

Moreover, in our case, the cysC remained stable at the baseline level in repeat analysis. However, there are still a few circumstances associated with cysC mismeasurement removing that biomarker out of an exclusively unfailing state. There is broad evidence that cysC is relevantly influenced by thyroid function. Patients with a hyperthyroid state commonly display higher cysC levels, whereas patients with a hypothyroid state tend to present with lower cysC levels. Therefore, in patients with thyroid dysfunction, the use of cysC for renal assessment should be avoided (*15*). Apart from thyroid hormones, corticosteroids can also be responsible for false cysC elevations without reflecting the actual renal state (*16*). In this context, it should be mentioned that cysC and creatinine alterations also affect the corresponding respective eGFR results. In our case, cysC mismeasurements due to the aforementioned causes could be excluded because our patient had neither endocrinological diseases nor had taken medications affecting the corticotropic or thyrotrophic axis and decisively obtained non-pathological results in both cysC analyses.

## What you can do in your laboratory to prevent such errors

Postprandial laboratory assessment of renal status should be performed by the analysis of cysC with subsequent eGFR calculation instead of creatinine measurement. In elective settings, blood samples should be taken after an appropriate fasting time of 12 hours. Furthermore, repeat testing should not be performed without a reflected indication.
